# Contamination of Consensus Sequences in Next-Generation Mitogenomics: The Published Mitochondrial Genome of *Haliastur indus* Is a Chimera with DNA from *Butastur indicus*. Comment on Sonongbua et al. Insights into Mitochondrial Rearrangements and Selection in Accipitrid Mitogenomes, with New Data on *Haliastur indus* and *Accipiter badius poliopsis*. *Genes* 2024, *15*, 1439

**DOI:** 10.3390/genes17010087

**Published:** 2026-01-14

**Authors:** George Sangster, Jolanda A. Luksenburg

**Affiliations:** 1 Naturalis Biodiversity Center, Darwinweg 2, 2300 RA Leiden, The Netherlands; 2 Institute of Environmental Sciences, Leiden University, Einsteinweg 2, 2333 CC Leiden, The Netherlands; 3 Department of Environmental Science and Policy, George Mason University, 4400 University Drive, Fairfax, VA 22030 4444, USA

The quality and authenticity of DNA sequences have long been points of concern for molecular biologists and systematists [1,2,3]. As the field of mitogenomics moves from traditional (Sanger) sequencing to next-generation sequencing (NGS), new challenges may arise and old problems may present themselves in new ways. While multiple chimeric mitogenomes that were generated using Sanger sequencing have been documented [4,5,6,7,8,9,10,11], so far no chimeras appear to have been identified that were generated using NGS. Here we provide evidence that a recently published mitochondrial genome that was generated using NGS is an interspecific chimera, demonstrating that this is likely an overlooked problem in next-generation mitogenomics.

A complete mitochondrial genome of the Brahminy Kite *Haliastur indus* (Aves: Accipitridae) was published as GenBank accession number OP133375.1/NC_066800.1 and documented, along with a mitochondrial genome of *Accipiter badius*, in a detailed paper in *Genes* [12]. The DNA sequence was generated using an Illumina NovaseqTM 6000 from a blood sample of a live bird obtained in Samut Prakarn, Thailand. This is the first mitogenome of *H. indus* and thus has the potential to become the reference sequence for future mitochondrial DNA applications.

We assessed the phylogenetic placement of OP133375.1 among Accipitriformes using three mitochondrial markers that are commonly used in avian phylogenetics: NADH dehydrogenase subunit 2 (*ND2*, 1041 bp), part of cytochrome oxidase subunit 1 (*CO1*, 696 bp), and cytochrome b (*CYB*, 1143 bp). The MITOS2 web server [13] was used to obtain information on the first and last positions of individual genes. MUSCLE [14] (as implemented in MEGA7 [15]) was used to align sequences. Maximum Likelihood phylogenies were obtained using IQ-tree version 2.2.2.6 [16]. We partitioned the data sets by codon. The appropriate substitution model for each partition in the data sets was selected using ModelFinder [17]. Branch support was obtained using 1000 ultra-fast bootstraps [18]. We used a sequence of *Cathartes melambrotus* (PQ153910, [19]) as an outgroup based on previous studies of Accipitriformes [20,21]. Sequence divergence was calculated as uncorrected *p*-values with complete deletion of nucleotide positions with missing data.

Following [6], we looked for evidence of chimeras based on one or more of five possible signs: (i) placement on a long branch, (ii) a deep divergence, (iii) a distant position from its expected phylogenetic position, (iv) a sister species placed on a long branch, (v) a mismatch between gene trees. An anomalous sequence was visually inspected and compared with sequences of conspecifics to localize any divergent fragment(s).

In the *ND2* gene tree, OP133375.1 was sister to a reference sequence of *H. indus* with 100% bootstrap support but the two sequences were rather divergent (as shown by their relatively long branches; Figure 1a). Uncorrected sequence divergence between the two sequences was 3.7%. In the *CO1* gene tree, OP133375 clustered with two reference sequences of *H. indus* with a very small sequence divergence (0.0–0.3%; Figure 1b). In the *CYB* gene tree, OP133375.1 clustered with three reference sequences of *Butastur indicus* with high bootstrap support (100%; Figure 1c). The *CYB* fragment of OP133375.1 was identical to that of one of the *B. indicus* sequences (AB830616), and differed from two other reference sequences by 0.5–0.6%. Sequence divergence between OP133375.1 and a single *CYB* reference sequence of *H. indus* (AY987309) was 10.9%.

A large fragment of OP133375.1 was identical to one of the *B. indicus* sequences (AB830616) and differed 1.3% from another mitogenome of *B. indicus* (MW017133). This fragment comprised 8994 bp and spanned the positions 1–1690 and 11,752–19,055 of OP133375; this corresponded to 47.2% of the total length of the mitogenome.

Our data show that OP133375.1 was present at two different well-supported phylogenetic positions in mitochondrial gene trees. This is a clear indication that this sequence is a chimera comprising DNA from at least two different species. The *CO1* gene tree indicates that part of the mitogenome is referable to *H. indus* and the *CYB* gene tree shows that the mitogenome also includes DNA fragments of *B. indicus*. Our data further show that a long fragment of mitogenome OP133375.1 is identical to a mitogenome sequence of *B. indicus*. Consequently, mitogenome OP133375.1 is not an accurate representation of the true mitogenome of *H. indus* and should not be used in biological studies.

Given that the mitogenome was obtained with NGS technology [12], the only plausible explanation for the observed sequence abnormalities is that these are due to failure to remove a reference sequence (almost certainly *B. indicus* AB830616) during the preparation of the consensus sequence.

Our study shows, perhaps for the first time, that chimeras do occur in mitogenomes assembled from NGS-generated data. Whereas in traditional (Sanger) sequencing work chimeras result from errors during the ‘wet’ phase (i.e., in the laboratory due to the inadvertent transfer of a DNA extract or PCR product to the wrong tube), NGS chimeras may be generated in the ‘dry’ phase (i.e., while generating a consensus sequence during the bioinformatics phase of the study).

The cladogram in [12] showed *H. indus* in its expected phylogenetic position, i.e., sister to Black Kite *Milvus migrans* [20,21]. This is quite remarkable given that almost 50% of its sequence is derived from a more distantly related species. Thus, a chimera may be difficult to detect in a phylogeny that includes no other conspecific sequences. We were able to detect this chimera because we generated separate gene trees of three markers, and because the problematic parts of the mitogenome referred to another, not particularly closely related species, and overlapped with one of the three reference markers. The detection of chimeras will be almost impossible if (i) a reference sequence of a conspecific individual is used, or (ii) the heterospecific fragment(s) do not overlap with at least one of the reference markers.

Large numbers of mitogenomes are now routinely generated for a single study using NGS technology [22,23,24,25,26,27]. The generation of the consensus sequence may be regarded as the Achilles heel of NGS mitogenomics because a seemingly small error (failure to remove the reference sequence) may have far-reaching consequences. Awareness of the problem of chimeras is important because chimeras may compromise research studies, including studies that re-use one or more mitogenomes for other purposes. For instance, previously published chimeric sequences have led to proposals for unwarranted taxonomic revision and may have compromised comparative analyses and measurements of divergence times [6]. We hope that our commentary will draw attention to the risk of generating chimeric sequences and will help others detect and document additional cases of this likely overlooked problem.

## Figures and Tables

**Figure 1:**
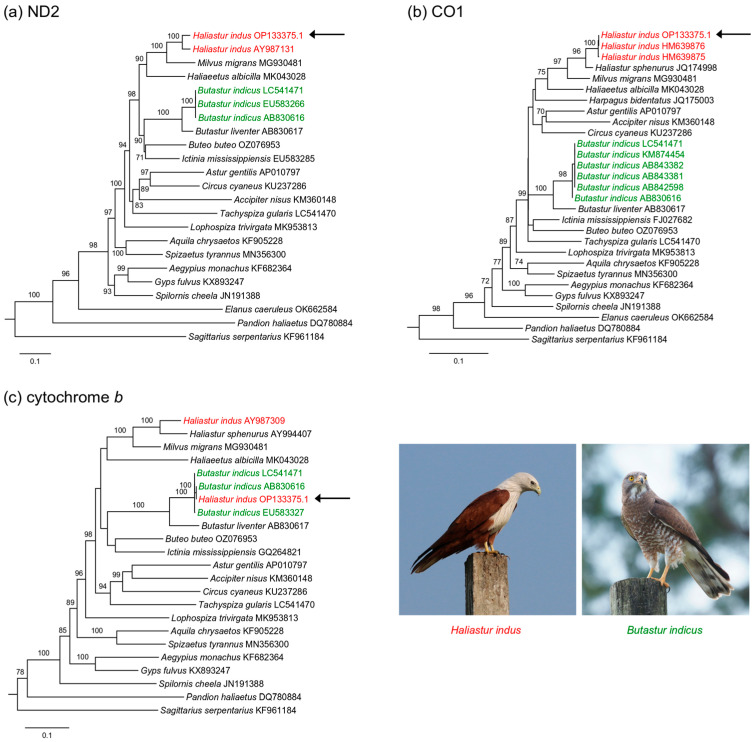
Maximum Likelihood phylogenies of Accipitriformes based on (**a**) *ND2* (1041 bp), (**b**) *CO1* (696 bp), and (**c**) *CYB* (1143 bp) sequences. The outgroup (*Cathartes melambrotus*) is not shown. Numbers along branches represent bootstrap support values (>70%) based on 1000 pseudoreplications. The arrows indicate the problematic sequence of *Haliastur indus*. Photograph of Brahminy Kite (*Haliastur indus*) by Shino Jacob Koottanad (CC-BY-SA 4.0, Wikimedia); photograph of Grey-faced Buzzard (*Butastur indicus*) by nhm6306 (CC-BY-NC, iNaturalist).
